# Integrative Metabolomic and Transcriptomic Analyses Reveal Metabolic Changes and Its Molecular Basis in Rice Mutants of the Strigolactone Pathway

**DOI:** 10.3390/metabo10110425

**Published:** 2020-10-26

**Authors:** Xiujuan Zhou, Ling Liu, Yufei Li, Kang Li, Xiaoli Liu, Junjie Zhou, Chenkun Yang, Xianqing Liu, Chuanying Fang, Jie Luo

**Affiliations:** 1 College of Tropical Crops, Hainan University, Haikou, Hainan 570288, China; xiujuan_1994@163.com (X.Z.); ling.liu@hainanu.edu.cn (L.L.); lk900109@163.com (K.L.); xiaoliliu1009@163.com (X.L.); sydlxy_0327@foxmail.com (J.Z.); liuxq@hainanu.edu.cn (X.L.); 2 National Key Laboratory of Crop Genetic Improvement and National Center of Plant Gene Research (Wuhan), Huazhong Agricultural University, Wuhan 430070, China; liyufeia615@foxmail.com (Y.L.); victoryang@webmail.hzau.edu.cn (C.Y.)

**Keywords:** strigolactones, metabolome, transcriptome, defense, WRKY45, rice (Oryza sativa)

## Abstract

Plants have evolved many metabolites to meet the demands of growth and adaptation. Although strigolactones (SLs) play vital roles in controlling plant architecture, their function in regulating plant metabolism remains elusive. Here we report the integrative metabolomic and transcriptomic analyses of two rice SL mutants, *d10* (a biosynthesis mutant) and *d14* (a perception mutant). Both mutants displayed a series of metabolic and transcriptional alterations, especially in the lipid, flavonoid, and terpenoid pathways. Levels of several diterpenoid phytoalexins were substantially increased in *d10* and *d14*, together with the induction of terpenoid gene cluster and the corresponding upstream transcription factor *WRKY45*, an established determinant of plant immunity. The fact that *WRKY45* is a target of *IPA1*, which acted as a downstream transcription factor of SL signaling, suggests that SLs contribute to plant defense through *WRKY45* and phytoalexins. Moreover, our data indicated that SLs may modulate rice metabolism through a vast number of clustered or tandemly duplicated genes. Our work revealed a central role of SLs in rice metabolism. Meanwhile, integrative analysis of the metabolome and transcriptome also suggested that SLs may contribute to metabolite-associated growth and defense.

## 1. Introduction

Since the first proto-life forms emerged on Earth some four billion years ago, a myriad of metabolites has been produced in a series of organisms to meet the demands for energy, growth and development, and for adaptation to the environment. Plants, which are evolutionarily forced to adapt to a constantly changing environment due to their sessile lifestyle, have been estimated to produce approximately 0.1 to 1 million phytochemicals [1,2]. Various kinds of metabolites have been shown to contribute to plant defense against phytopathogenic microorganisms. These include phytoalexins derived from terpenoid, flavonoid, and phenylamide metabolism [3].

The wide array of plant metabolites represents a world of rich biological complexity and significance [4,5]. Flavonoids, synthesized by a combination of the phenylpropanoid and acetate-malonate metabolic pathways, provide an example of this complexity and significance. The core structures of flavonoids are produced by successive reactions catalyzed by multiple enzymes, including chalcone synthases (CHS), chalcone isomerases (CHI), hydroxylases, reductases, and oxidases [6,7,8]. The core flavonoids are subjected to multiple modifications, such as hydroxylation, oxidation, methylation, glycosylation, and acylation. Frequently occurring tailoring modifications contribute greatly to the structural as well as functional diversity of flavonoids [9,10,11,12,13] with dozens of flavonoids detected in most plant species, showing significant interspecies and intraspecies diversity [11,14,15,16,17].

Various evolutionary routes to biochemical innovation have been adopted in plants, one of which is the use of biosynthetic gene clusters [18,19,20,21,22]. Using a computational pipeline, approximately 12,000 metabolic gene clusters were predicted from 18 species [23]. There are several examples of metabolic pathways controlled by regulon-like gene clusters, such as the terpenoids and alkaloids [24]. Diterpenoids, which comprise a considerable proportion of the terpenoid complement, originate from consecutive reactions mediated by mechanistically distinct, yet phylogenetically related, diterpene synthases (DiTPSs), including the labdadienyl/copalyl diphosphate synthase (CPS) and the kaurene synthase (KS) belonging to class II and class I DiTPSs, respectively. Various diterpene scaffolds are further modified by heme-thiolate cytochrome P450 monooxygenases to produce bioactive compounds, such as oryzalexins, momilactones, oryzalides, phytocassanes, and GAs in rice [25]. There are two well-established biosynthetic gene clusters of diterpenoids in the rice genome, containing genes encoding CPSs, KSs, and P450s [26]. To date, transcription factors from different families have been indicated to regulate the expression of diterpene gene clusters [27,28,29]. For instance, *WRKY45*, which is essential for rice resistance to various biotic and abiotic stresses [30,31,32], has been documented to play an essential role in priming diterpenoid phytoalexin biosynthesis by regulating biosynthetic gene clusters [27].

In addition, gene duplication and divergence also contribute to the evolution of plant metabolic diversity, as evidenced by evolutionary metabolomics [16,33,34,35,36]. A quantitative trait locus (QTL) study on the biosynthesis of strigolactones (SLs) also supports the involvement of gene duplication in metabolic diversification [37]. SLs, a class of sesquiterpene lactones, are downstream products of carotenoid catabolism. The precursor of SLs, namely carlactone, is produced by successive reactions catalyzed by DWARF27 (D27), carotenoid cleavage dioxygenase 7 (CCD7), and CCD8 (also denoted as D10 in rice) [38]. Then, carlactone is oxidized by the cytochrome P450 monooxygenase MORE AXILLARY GROWTH 1 (MAX1/CYP711A1) in *Arabidopsis* [39,40]. There are five orthologues of *MAX1* in rice, three of which (denoted as *CYP711A2*-*4*) are tandem duplicates on the first chromosome [41]. *CYP711A2* and *CYP711A3* (also known as *Os900* and *Os1400*) share convergent functions in the conversion of carlactone to carlactonoic acid, while *CYP711A4*/*Os1500* is nonfunctional due to a premature stop codon [40]. Despite the close phylogenetic relatedness of their protein sequences, these two duplicates also display divergent roles in further reactions [40,42]. Additionally, the transcriptional response of *CYP711A2* to phosphate deficiency is also distinct from that of *CYP711A3* [37]. Moreover, the tandemly repeated *P450s* on chromosome 1 also respond to the variation in SL content between *indica* and *japonica*, two major subspecies of rice [37]. This indicates that the SL pathway may have undergone natural or artificial selection. Encouraging evidence for this hypothesis also comes from a study on *Ideal Plant Architecture 1* (*IPA1*), which encodes a SQUAMOSA promoter binding protein-like (SPL) transcription factor downstream of SL signaling [43]. *IPA1* contributes to balanced growth and immunity in rice [44], which harbors functional genetic variants across accessions [45,46].

A myriad of efforts has been made to illustrate the function of SLs in overall plant architecture [47]. Despite intensive previous work, the exact role of SLs in plant metabolism remains to be elucidated. Herein, we performed metabolomic and transcriptomic profiling with rice mutants of SL biosynthesis and signaling, namely, *d10* and *d14*. Compared to the wild-type (WT) plants, a series of metabolic and transcriptional alternations were identified in the two mutants, especially in the lipid, flavonoid, and terpenoid pathways. The defective SL pathway also led to the altered accumulation of different kinds of diterpenoid phytoalexins. Notably, *WRKY45* and the biosynthetic gene cluster of diterpenoids under its control displayed altered transcript levels in *d10* and *d14*, suggesting that SLs may contribute to plant immunity through *WRKY45* and phytoalexins. Moreover, we also found that SLs regulate metabolism via genes with evolutionary signatures.

## 2. Results

### 2.1. Metabolomic Analysis of SL Mutants and WT Plants

**Figure 1:**
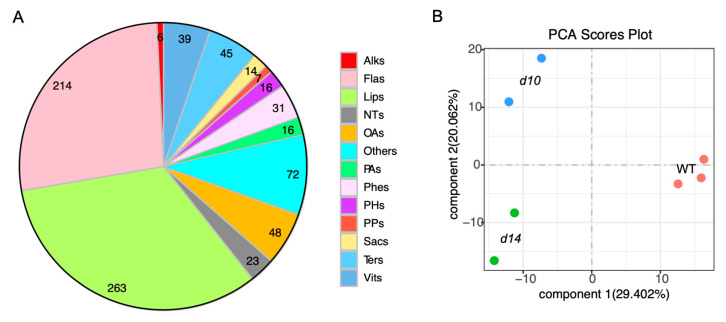
Metabolomic analysis of SL mutants and wild-type (WT) plants. (**A**) A total of 794 metabolites were detected in this study. Alks, alkaloids; Flas, flavonoids; Lips, lipids; NTs, nucleic acids and nucleotide derivatives; OAs, organic acids; Phes, phenolamines; PHs, phytohormones; PAs, polyamines; Sacs, saccharides; Ters, terpenoids; Vits, vitamins. (**B**) Principal component analysis (PCA) of the 794 metabolites in *d10*, *d14*, and WT. PC1 and PC2 refer to the first and second principal components, respectively. *d10* and *d14* represent mutants of *DWARF10* and *DWARF14*, which are essential in the biosynthesis and signal transduction of SLs, respectively. WT refers to the background of the mutants, namely, ZH11.

To capture a wide range of metabolic responses to SLs in rice, a previously established and developed widely targeted metabolomics method [48] was used with the WT and previously created biosynthesis and perception mutants, namely, *d10* and *d14* [49]. The leaves of each genotype from the rice plants grown with hydroponic culture were sampled and extracted. The extracts were then subjected to an HPLC-ESI-MS/MS analysis. In total, 794 compounds were detected (Table S1). These included both primary and secondary metabolites: (i) the majority of the primary metabolites were derived from lipid metabolism, such as phosphatidylcholines (PCs) and lysophosphatidylcholines (lysoPCs); (ii) various pathways were associated with the secondary metabolites detected, including 214 flavonoids and 45 terpenoids (Figure 1A). The metabolomic data of the WT and mutants were clustered into three distinct groups after an unsupervised principal component analysis (PCA) of all the detected metabolites with log10 values (Figure 1B). The PCA showed that the first two principal components (PC1 and PC2) accounted for approximately 49% of the total differences among the three genotypes (Figure 1B).

**Figure 2:**
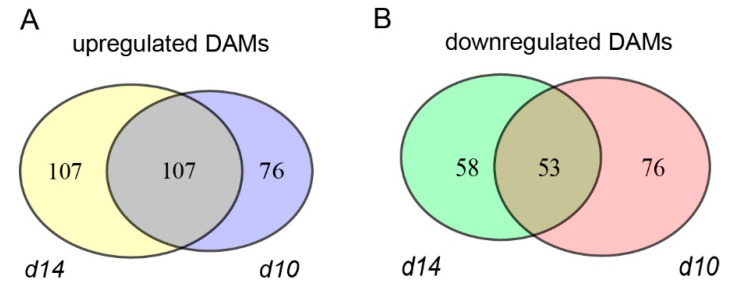
Schematic representation of metabolites with altered accumulation levels in the mutants. The number of up-regulated (**A**) and down-regulated (**B**) DAMs (differentially accumulated metabolites) in *d10* and *d14*.

To reveal the metabolic divergence between the mutants and WT in-depth, differentially accumulated metabolites (DAMs) were identified (fold change > 1.5 times). First, we compared the metabolomic data of the WT with those of *d10* and *d14*. In total, 183 and 129 metabolites accumulated at relatively higher and lower levels, respectively, in *d10* leaves than in WT leaves (Figure 2). Further data mining revealed that most of the top 10% of the DAMs between the *d10* and WT plants were classified into flavonoids, lipids, and phenolamines (Table S2). For the *d14* leaves, the numbers of up- and down-regulated metabolites were 214 and 111, respectively (Figure 2). Metabolites from phenolamines, flavonoids, and lipids accounted for a majority of the top 10% of DAMs in *d14*, consistent with the result for *d10* (Table S2). Next, we compared DAMs in *d10* and *d14* to define the SL-modulated metabolites. Overall, 190 DAMs were commonly found in the biosynthesis and perception mutants of SLs, most of which were flavonoids and lipids (Table S2).

### 2.2. Transcriptome Profiling of the Leaves from WT and SL Mutants

To obtain a molecular interpretation of the metabolic responses in the SL mutants, Illumina RNA-sequencing-based transcriptome profiling was conducted. A total of approximately 264 million clean reads were derived, with an average of 97% that could be mapped to the reference genome of rice (Table S3). RNA reads from each sample were then aligned to the well-annotated genome of Nipponbare (MSU 7.0). Subsequently, the relative expression of each gene was calculated using fragments per kilobase of exon per million fragments mapped (FPKM) values. Our further interpretation is mainly restricted to the genes with a mean FPKM ≥ 1 in at least one genotype. Prior to deeper mining of the transcriptomic data, the repeatability of the data from independent biological replicates was confirmed by correlation analysis (Figure S1).

To test the reliability of our transcriptomic data, we checked genes under the well-validated regulation of SLs. The expression of *D10* is significantly induced in the two mutants (Figure S2A), which is consistent with the reported negative feedback regulation by SLs of its orthologues in *Arabidopsis* [50]. Then, a quantitative real-time polymerase chain reaction (qRT-PCR) assay was carried out and validated that *D10* and *D14* expressed at significantly higher levels in the mutants (Figure S2B).

Additional evidence was obtained from the observation that two auxin efflux transporter encoding genes, namely, *PIN1a* (LOC_Os06g12610) and *PIN1b* (LOC_Os02g50960), displayed elevated transcript abundance in the two mutants (Figure S2A). This is consistent with the repression effect of exogenous SL analogs on the expression of *PIN1a* and *PIN1b* [51].

**Figure 3:**
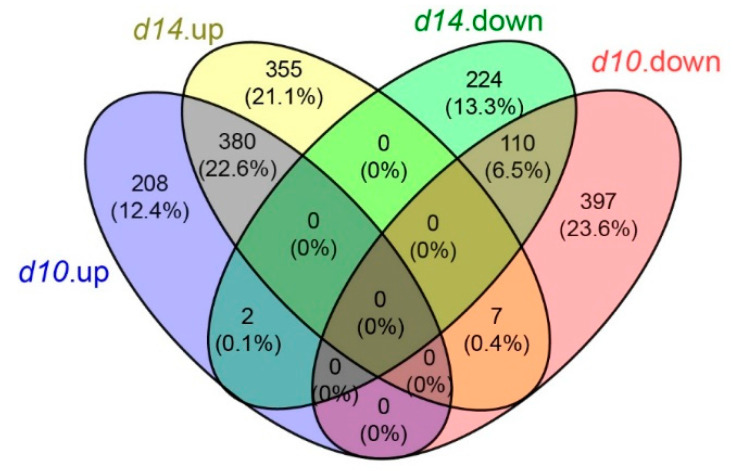
Summary of the differentially expressed genes (DEGs) in *d10* and *d14.* Venn diagrams showing the number of significantly up-regulated and down-regulated genes that were uniquely or commonly regulated in the mutants.

To identify genes affected by defective SL pathways, the differentially expressed genes (DEGs) were identified, considering a two-fold expression change as the cut-off with a *p*-value < 0.05. A total of 1104 and 1078 DEGs were identified in *d10* and *d14*, respectively, with more than half up-regulated (i.e., ~53.4% in *d10* and ~68.6% in *d14*) (Table S4). A combined analysis of DEGs in *d10* and *d14* led to the observation that 380 and 110 genes were up- and down-regulated, respectively, in common, while a considerable number of genes were uniquely differentially expressed as well (Figure 3).

**Figure 4:**
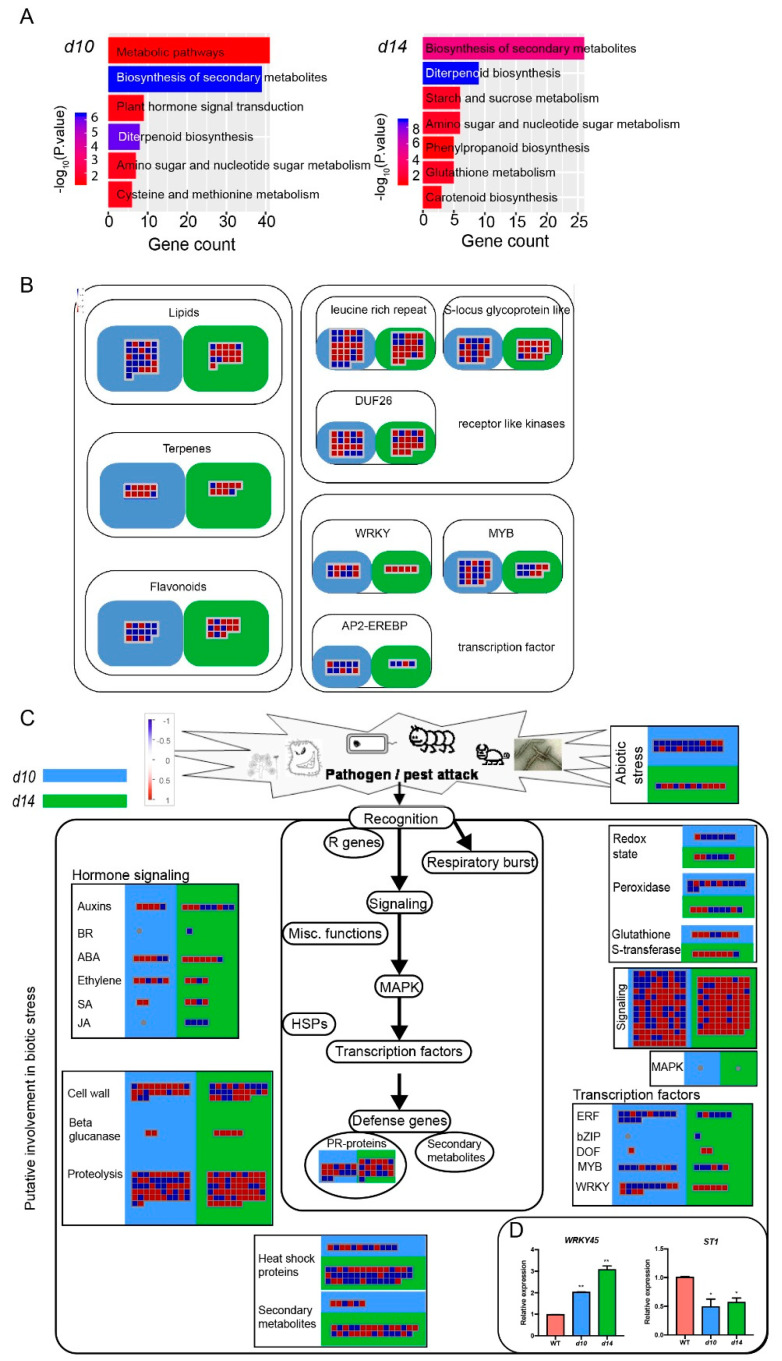
MapMan and KEGG analysis of the DEGs in the mutants. (**A**) Kyoto Encyclopedia of Genes and Genomes (KEGG) analysis of the differentially expressed genes (DEGs) in *d10* vs. WT and *d14* vs. WT. (**B**) Overview of the secondary metabolism-related DEGs in the mutants. (**C**) Enrichment of DEGs into biotic stress pathways in *d10* (blue rectangle) and *d14* (red rectangle). In the heat maps in B and C, red and blue indicate upregulation or downregulation in the mutants compared with the WT, respectively. Colored boxes in each region represent multiple variations in gene expression. (**D**) qRT-PCR based expression levels of *WRKY45* and *ST1* in *d10*, *d14*, and WT. The data are represented as mean ± SD of two biological replicates. The Student’s *t*-test analysis indicates a significant difference (compared with WT, * *p* < 0.05, ** *p* < 0.01).

Next, the Kyoto Encyclopedia of Genes and Genomes (KEGG) analysis was performed with the DEGs in *d10* and *d14*. We found that ten pathways were significantly enriched in *d10* vs. WT and *d14* vs. WT, including three shared by *d10* and *d14*, namely, biosynthesis of secondary metabolites, diterpenoid biosynthesis, and amino sugar and nucleotide sugar metabolism (Figure 4A). To investigate the effect of SLs on the expression of metabolic genes in detail, we used MapMan 3.6.0 software for further analysis. The MapMan-based analysis of the secondary metabolism overview showed that the various metabolic genes were regulated by SLs. Of note were genes participating in the terpenoid pathway and phenylpropane-derived metabolism (Figure 4B). Furthermore, the DEGs of *d10* and *d14* were also mapped onto biotic stress pathways with MapMan (Figure 4C). Consistent with the KEGG results, genes corresponding to secondary metabolites were found to be enriched with altered expression levels. In addition, we observed that genes classified into the following categories presented significant enrichment: signaling, proteolysis, cell wall, PR-proteins, and transcription factors. Notably, a well-documented transcription factor denoted as *WRKY45* displayed activated expression in *d10* and *d14* (Figure 4D). There are two natural alleles of *WRKY45* in rice, playing opposite roles in rice resistance to *Magnaporthe oryzae* [52]. The *japonica*-derived allele *WRKY45-1* negatively regulates resistance to blast disease, and the first intron of this allele contains the transposon-derived small RNA *TE-siR815*. *TE-siR815* suppresses the leucine-rich repeat receptor kinase encoding *ST1* (LOC_Os08g10150), which is an important component in *WRKY45*-mediated resistance [34]. The SL mutants and WT plants used in this study carry the *japonica* type *WRKY45-1*. Along with the increased transcript abundance of *WRKY45* in *d10* and *d14*, the expression levels of *ST1* in the SL mutants were reduced to approximately 25% of that in WT, albeit with a low statistical significance (Figure 4D). This observation is similar to that of studies on transgenic plants overexpressing *WRKY45-1* [34]. Therefore, it is conceivable that SLs may contribute to plant defensive actions involving fine-tuned regulation and signal transduction.

### 2.3. SLs Regulate Diterpenoid Phytoalexins via WRKY45-Modulated Clustered Genes

**Figure 5:**
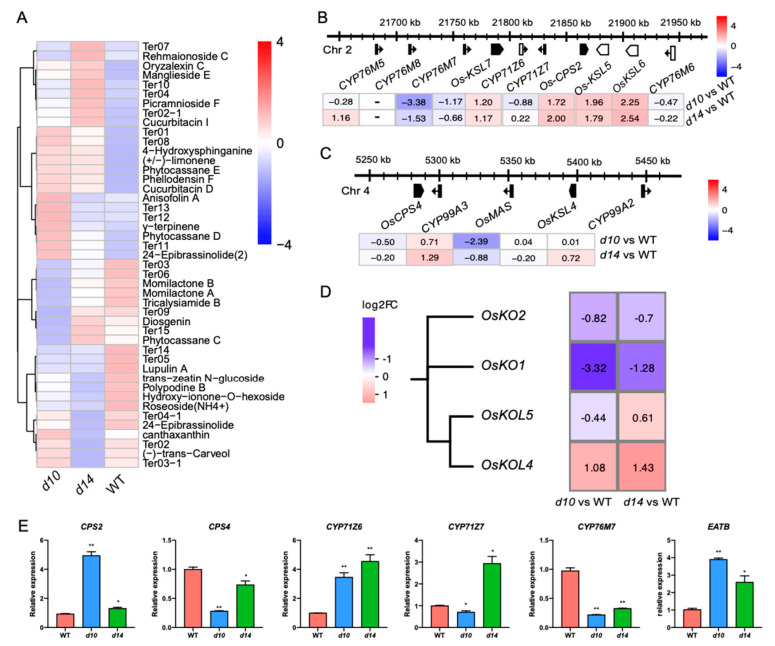
SLs regulate terpenoids via clustered or tandemly duplicated genes. (**A**) Metabolic profiling of terpenoids in *d10*, *d14*, and WT. Heat map visualized with the average content of terpenoids after normalization in at least two biological replicates. The expression of genes in the CPS2 cluster (**B**) and CPS4 cluster (**C**) in the mutants. In (**B**) and (**C**), the schematic diagram represents the CPS2 and CPS4 clusters, with the arrowheads representing the direction of transcription. (**D**) The members of the ent-kaurene oxidase-encoding family and their transcriptional responses in the mutants. In (**B**–**D**), the altered expression level of each gene is represented by log_2_(FC). Red and blue represent upregulation and downregulation, respectively. (**E**) qRT-PCR based expression levels of *CYP71Z6*, *CYP71Z7*, *CYP76M7*, *CPS4*, *CPS2*, and *EATB* in *d10*, *d14*, and WT. The data are represented as mean ± SD of two biological replicates. The Student’s *t*-test analysis indicates a significant difference (compared with WT, * *p* < 0.05, ** *p* < 0.01).

Of the 45 structurally identified or annotated terpenoids, 10 were identified as DAMs in both of the mutants, and 60% of these DAMs were up-regulated (Figure 5A). Notably, we found that the leaves of *d10* and *d14* displayed enhanced accumulation of diterpenoid phytoalexins, including phytocassane D, phytocassane E, and oryzalexin C. This is consistent with the observation that genes related to secondary metabolites were enriched in the MapMan mapping onto the biotic stress pathways. This finding also supports the view that SLs may contribute to plant defense.

To explore the molecular basis of the SL-regulated production of terpenoids, we re-inspected the transcriptome profile. Given that successive cyclization of geranylgeranyl diphosphate catalyzed by CPSs and KSs is essential for producing diterpenoids, we analyzed the expression of the encoding genes. *CPS2*, *KSL5*, and *KSL6* were induced significantly in *d10* and *d14*, with a more than 3.2-fold change in expression level in each genotype compared with the level in WT (Table S5). This is consistent with the altered accumulation levels of phytoalexins in SL mutants, considering the previously reported function of *CPS2*, *KSL5*, and *KSL6* in the biosynthesis of diterpenoids. Notably, *CPS2*, *KSL5*, and *KSL6* have been described to be part of a key gene cluster (referred to as the *CPS2* cluster hereafter) involved in the synthesis of diterpenoids [28,29]. This cluster consists of *CPS2*, three *KSLs* (*KSL5*–*7*), and six *P450s* (two from the CYP71Z family and four from the CYP76M family). The clustered *P450s* have been characterized to be co-regulated with *CPS2* and *KSL7*, which is a feature of gene clusters [26,53]. Reasonably, this feature raised the question of whether SLs exert effects on the expression of the whole *CPS2* cluster. To address this issue, we analyzed the expression pattern of the *P450s*, with the exception of *CYP76M8*, due to its relatively low abundance (mean FPKM less than one in each sample). *CYP71Z6* was expressed at ~2.2-fold higher levels in the SL mutants than in the WT. The expression level of *CYP76M7* in *d10* and *d14* was reduced to less than half of that in WT (Figure 5B). That is, a majority of the *CPS2* clusters are under the control of SLs. In addition to the *CPS2* cluster, there is another biosynthetic gene cluster of diterpenoids on chromosome four (referred to as the *CPS4* cluster hereafter), which consists of *CPS4*, *KSL4*, a dehydrogenase gene (*MAS*), and two *P450s* belonging to the CYP99A family [26]. An expression analysis was performed with *MAS* and *CYP99A* members from the *CPS4* cluster, even though there was no significant difference in the expression of *CPS4* or *KSL4* in the SL mutants and WT (Figure 5C). Mutations in *D10* and *D14* triggered the expression of *CYP99A3*, while *CYP99A2* was not significantly affected (Figure 5C). The expression of the DEGs we have mentioned above was verified by qRT-PCR (Figure 5E).

Clustered genes tend to be coordinately regulated by transcription factors, which makes it possible for plants to respond to external stimuli immediately. Of additional interest is the identification of transcription factors with potential roles in SL-regulated biosynthetic gene clusters of diterpenoids. To this end, we explored the expression pattern of transcription factors that have been shown to modulate the aforementioned clusters. As described above, *WRKY45*, which has also been reported to mediate the expression of *CPS2* and *CPS4* cluster genes, was expressed at more than 2.1 times higher levels in both mutants than in the WT (Figure 4D).

To conclude, SLs modulate diterpenoid phytoalexin metabolism via biosynthetic gene clusters, possibly through *WRKY45*, which suggests the involvement of SLs in plant defense.

### 2.4. SLs Modulate a Series of Clustered or Tandemly Duplicated Genes

These findings prompted us to assess the effects of SLs on metabolic gene clusters from the whole genome. Hence, we analyzed the expression pattern of genes included in 793 metabolic gene clusters predicted by Schlapfer et al. (Supplementary Table S4) [23]. Overall, 57 clusters were marked as being SL regulated, of which at least two genes displayed a greater than two-fold change in each mutant (with similar tendencies). The proportion of the genes that responded to SLs in each cluster varied from approximately 5% to 41%, with an average of approximately 20% (Table S6). In addition, the predicted gene cluster C312_4, covering the *CPS2* cluster, was also selected as being SL regulated. Notably, C645_4 is also regulated by SLs, containing four tandemly duplicated *P450s* with ent-kaurene oxidase activity in diterpenoid biosynthesis, namely, *KO1*, *KO2*, *KOL4*, and *KOL5*. *KOs* and *KOLs* displayed divergent responses to mutations of SLs, regardless of their phylogenetic relatedness. *KOL4* was expressed at approximately 2.1-fold to 2.7-fold higher levels in SL mutants than in WT (Figure 5D). The expression level of *KO1* in *d10* and *d14* was reduced to less than half of that in WT (Figure 5D). Additionally, in *d10* and *d14* leaves, the abundance of the transcript of *KO2* was repressed to no more than 61% of that in WT plants, despite a low significance (Figure 5D). In other words, SLs regulate the biosynthetic gene cluster of diterpenoid, including several genes catalyzing GAs synthesis. Additional evidence supporting SL-modulated GA production came from the observation that several genes involved in GA biosynthesis or catabolism are regulated by SLs. For instance, *GA20ox1* and *GA2ox10* were significantly repressed in the mutants (Figure S3). In addition, we noticed that the AP2/ERF factor-encoded gene *EATB*, a negative regulator of GA synthesis [54], was significantly induced in *d10*, with a more than five-fold increase in transcript level compared with that in WT. Meanwhile, the expression level of *OsEATB* in *d14* was approximately 1.7-fold higher than that in WT (Figure S4). In summary, SLs affect GA-associated metabolism, at least partly via the convergent regulation of clustered genes and the divergent modulation of duplicated genes.

### 2.5. Affected Flavonoid Pathway in SL Mutants

**Figure 6:**
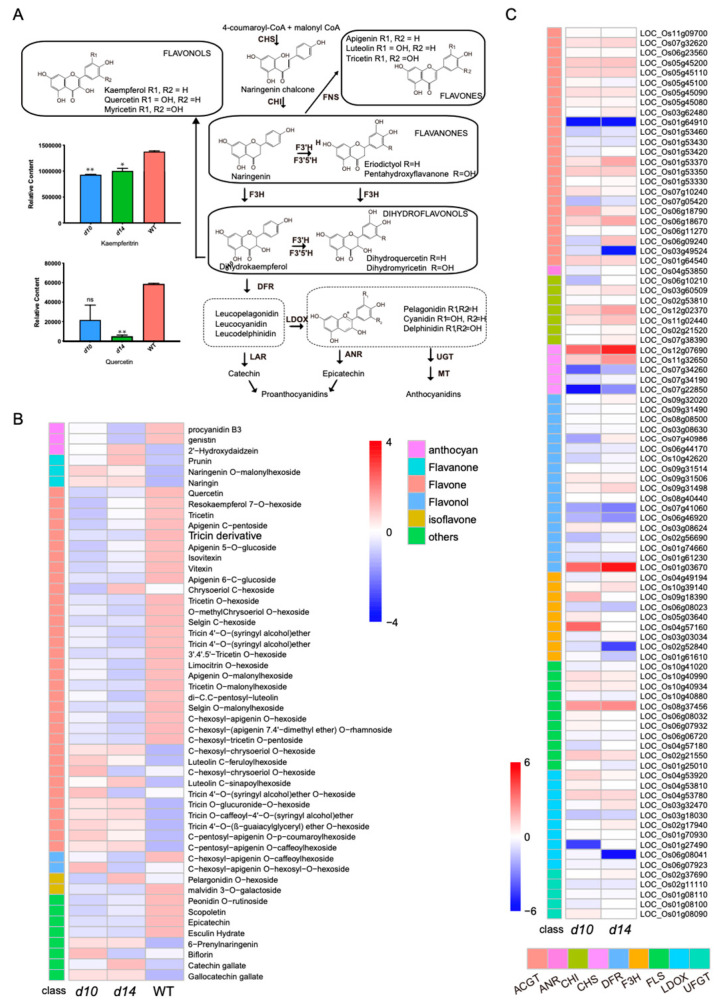
Affected flavonoid pathway in SL mutants. (**A**) Abundance of metabolites at the node of core flavonoid metabolism in *d10*, *d14*, and WT. (**B**) Heat map of flavonoids produced by tailoring reactions. The average content of each metabolite from individual biological replication of three genotypes was standardized and used to generate the heat map. (**C**) Heat map of the expression levels of genes with potential relatedness to the flavonoid pathway. The heat map was constructed with the average log_2_(FC) expression level in each mutant. ACGT: anthocyanidin 5 3-O-glucosyltransferase; ANR: anthocyanidin reductase; CHI: chalcone isomerase; CHS: chalcone synthase; DFR: dihydroflavonol-4-reductase; F3H: flavanone 3-hydroxylase; FLS: flavonol synthase; LDOX: leucoanthocyanidin dioxygenase; UFGT: UDP-glucuronosyl/UDP-glucosyltransferase family protein.

In addition to the terpenoid pathway, we focused on the effects of SLs on flavonoid metabolism, given that flavonoids constitute a vast proportion of secondary metabolites. First, we analyzed the abundance of metabolites at the node of core flavonoid metabolism. In the leaves of *d14* and *d10*, the level of quercetin (a typical structure of flavonols) was approximately 8% and 38%, respectively, of that in WT leaves (Figure 6A). Meanwhile, we also noticed a slight but significant decrease of <30% in the level of kaempferitrin in the mutants, which is another representative flavonol (Figure 6A). However, compounds with featured flavone structures, such as tricetin, luteolin, and apigenin, displayed almost no notable response in the two mutants. This means that the absence of SLs is likely to reduce the production of flavonoids, particularly the synthesis of flavonols. Subsequently, we analyzed the level of flavonoids produced by tailoring reactions. As shown in Figure 6B, 52 flavonoids displayed marked differences in content between the WT and mutants, with approximately 60% down-regulated.

To focus on how SLs influence genes involved in core flavonoid metabolism, we analyzed the expression of these genes according to the annotation from MSU (Figure 6C). Overall, a series of these genes displayed altered expression levels in *d10* and *d14*. In conclusion, defective SL pathways repressed the accumulation of flavonoids.

### 2.6. Effects of SLs on Lipid Metabolism

**Figure 7:**
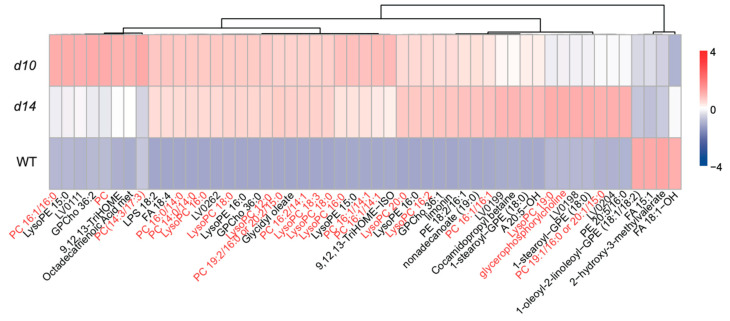
Effects of SLs on lipid metabolism. The relative content of lipids in *d10*, *d14*, and WT. The red font in the picture is choline related metabolites. LV0262, (23E)-23-(2-Diazonio-2,4-cyclopentadien-1-ylidene)-6,23-dihydroxy-2,2-dimethyl-11-oxo-9-[(stearoyloxy)-methyl]-5,7,10,22-tetraoxa-2-azonia-6-phosphatricosane 6-oxide; LV0204, 2-[(8-Carboxyoctanoyl)-oxy]-3-(palmitoyloxy)-propyl 2-(trimethylammonio)-ethyl phosphate; LV0199, (2S)-2-[(9-Oxononanoyl)-oxy]-3-(palmitoyloxy)-propyl 2-(trimethylammonio)-ethyl phosphate; LV0198, 2-[(7-Oxoheptanoyl)-oxy]-4-(palmitoyloxy)-butyl 2-(trimethylammonio)-ethyl phosphate; LV0111, 17-keto-4(Z),7(Z),10(Z),13(Z),15(E),19(Z)-Docosahexaenoic Acid.

To explore the effects of SLs on primary metabolism, we analyzed the accumulation of lipids in plants of each genotype. The majority of lipid species are classified into non-esterified fatty acids, sphingolipids, glycerolipids, and glycerophospholipids [55]. Glycerophospholipids could also be the targets of phospholipase D (PLD), which separates the polar moieties from the substrate to form phosphatidic acids (PAs) [56]. Overall, the levels of 47 DAMs classified as lipids were elevated in the two SL mutants, with the exclusion of 2-hydroxy-3-methylvalerate, 1-oleoyl-2-linoleoyl-GPE (18:1/18:2), FA 18:1-OH, and FA 15:1 (Figure 7). Notably, glycerophospholipid-related metabolites account for approximately 70% of the up-regulated DAMs classified as lipids. Thus, glycerophospholipid-related metabolism may be negatively regulated by SLs.

To explore the potential molecular basis of SL-regulated lipid metabolism, transcriptomic data were then further analyzed. In total, we identified four DEGs that may be involved in the glycerophospholipid pathway. Two genes encoding PLD were found to be induced in *d10* and *d14*, while a PLA-encoding gene was repressed (Figure S4). Serine also serves as the substrate to produce phosphoethanolamine, the precursor of phosphatidylcholines. Phosphoethanolamine is subjected to a three-step SAM-dependent methylation mediated by phosphoethanolamine *N*-methyltransferases (PEAMTs) [57]. We also characterized a PEAMT-encoding gene, named *PEAMT2* [58], that was down-regulated in the SL mutants (Figure S4). Taken together, our metabolomic and transcriptomic data show that SLs positively affect lipid metabolism.

## 3. Discussion

While our understanding of the association between SLs and various end phenotypes and the underlying mechanisms is growing, the role of SLs in metabolism and the way in which SLs contribute to adaptation via metabolites remain unknown. Herein, we report a combined analysis with transcriptomic and metabolomic data of rice mutants of the SL biosynthetic and signal transduction pathways. In this work, we discovered a series of metabolic responses to SLs in both primary and secondary metabolism, including in lipid, flavonoid, and terpenoid metabolism. Follow-up analysis of transcriptomic data showed the potential involvement of SLs in defensive reactions in rice. Furthermore, we found that SLs target clustered or tandemly duplicated genes to regulate GA-associated metabolism.

Based on the improved widely targeted metabolome strategy, 794 metabolites were detected in our work. In brief, lipids, terpenoids, and flavonoids display strong yet distinct changes in SL mutants. The majority of lipids and terpenoids with significant changes exhibited increased accumulation in *d10* and *d14*. The flavonoid pathway is apparently repressed in the two mutants. That is, the metabolic flux distribution is controlled by SLs, which may occur through an upstream switch. Glycerophospholipid-related metabolites accounted for the majority of the lipids with altered abundance in the mutants. It has been documented that phosphate deficiency leads to a reduced level of glycerophospholipids, as well as induced the accumulation of SLs [59]. In addition, glycerophospholipids are also involved in acclimation to limited phosphate in plants [60,61]. The altered content of glycerophospholipids in *d10* and *d14* could be a reflection of SL-mediated adaptation to phosphate deficiency.

Based on the transcriptomic and metabolomic data, we reasonably inferred a potential role of SLs in plant defense. Pieces of evidence underpinning this hypothesis arise from the observation that a series of genes, which are predicted to be of importance in defensive reactions, exhibited changed transcript abundances in the SL mutants. Notably, *WRKY45*, which is critical for plant defense [30,31,32], was also induced in the two mutants. Meanwhile, the vital component of *WRKY45*-mediated defense, namely, *ST1* [34], was repressed in *d10* and *d14*. This observation is reminiscent of the study of *WRKY45-1* overexpression lines, which also repress *ST1* and exhibit pathogen susceptibility. That is, SLs are likely to contribute to defensive reactions via *WRKY45*. This hypothesis is supported by further evidence from a study on *IPA1*, which has been identified as a key transcription factor downstream of SLs [43]. Infection with the fungus *Magnaporthe oryzae* (*M. oryzae*) activates the phosphorylation of IPA1, which can then bind to the *WRKY45* promoter and subsequently activate *WRKY45* expression. Consequently, phosphorylated IPA1 enhances the rice defense against *M. oryzae* [44]. In other words, SLs may contribute to enhanced immunity to biotic stress via *IPA1* and *WRKY45*.

Focusing on the terpenoid pathway, we found that diterpenoid phytoalexins accumulated at relatively high levels in *d10* and *d14*. These compounds are considered to be pivotal in plant immunity and are induced by microbial infections. Consistent with the metabolic responses of diterpenoid phytoalexins, biosynthetic genes, including *CPS2*, *KSL5*, *KSL6*, and *CYP71Z6*, were also activated in the mutants. This indicates the involvement of SLs in plant defense, considering the established function of phytoalexins in plant resistance to diseases. Notably, *WRKY45* has been shown to regulate a series of biosynthetic genes of diterpenoid phytoalexins [27], including some that were modulated by SLs in this work. As shown in our data, *WRKY45* is activated in *d10* and *d14*, subsequently inducing the expression of biosynthetic genes and the accumulation of phytoalexins. Wang et al. have revealed that GR24^4DO^ treatment induces *WRKY38*, which plays a role in defense responses in Arabidopsis [62]. That is in agreement with our findings. Nevertheless, the exact role of SLs remains to be illustrated, since induced phytoalexins enhance the plant defense in *d10* and *d14*, which is the opposite of the hypothesis that SLs contribute to enhanced immunity via *IPA1* and *WRKY45*.

Metabolic and functional diversification occurred during land plant evolution, benefiting from gene duplication and divergence [63]. It has been well documented that SLs are closely associated with various agricultural performance parameters in rice, such as tillering, plant height, flowering date, root architecture, and resistance to drought and salt [38]. Hence, the selection of hitchhiking genes in SL biosynthesis with genes controlling end phenotypes may account for the majority of the diversification in SL production and signal transduction. Evidence supporting this inference comes from the natural variation in SL levels and agricultural performance determined by the genetic divergence of *MAX1* in rice [37]. The evolution of SL biosynthesis and signaling, including gene duplication and divergence [37,40,64,65], led us to wonder whether SLs play a role in evolutionary metabolism. Biosynthetic gene clusters are remarkable hallmarks of plant metabolism compared to the genetic control of agricultural traits [66]. Herein, our work revealed that SLs regulate various metabolic gene clusters, including *WRKY45*-mediated clustered genes in the diterpenoid pathway. Moreover, we found that duplicated genes of GA biosynthesis divergently responded to defects in the SL pathway, providing additional evidence for the potential involvement of SLs in evolutionary metabolism.

Our work also provides new insights into the crosstalk of SLs with GAs, mutants of which in various cereals led to the green revolution [67]. GAs and SLs have been shown to regulate a largely overlapping set of genes in *Arabidopsis* [68]. Ito et al. (2017) reported that bioactive GAs repressed the production of SLs in rice in a GA signaling-dependent manner. Most of the biosynthetic genes of SLs are repressed by GAs [69]. Most recently, *D14* has been proven to be positively controlled by GAs via histone H3 lysine 27 trimethylation [67]. By and large, these studies clarified that GAs work upstream of SLs by regulating the biosynthesis and signal transduction of the latter. In this work, we identified transcriptomic evidence indicating that SLs, in turn, modulate the expression of genes involved in GA biosynthesis and homeostasis.

In conclusion, our work revealed an essential role of SLs in rice metabolism, coordinately modulating lipid, flavonoid, and terpenoid pathways. Meanwhile, we provide new insights into the involvement of SLs in rice immunity, which may occur through *IPA1*, *WRKY45*, and phytochemicals. In addition, we also identified the epistatic effect of SLs on GA biosynthesis, through which SLs may contribute to plant growth. The integrative analysis of the metabolome and transcriptome also suggested that SL-mediated metabolism may be a critical strategy evolved by plants to modulate growth and defense.

## 4. Materials and Methods

### 4.1. Plant Materials and Growth Conditions

Mutants of *D10* and *D14* were created in our previous work [49]. The rice plants used in this study were grown at Hainan University (Haikou, China, N 20°02′, E 110°11′). All the seeds were germinated for three days at 37 °C on filter paper soaked in distilled water and then planted in seedbeds. Two-week-old seedlings were subsequently planted by hydroponic culture using Yoshida nutrient solution as previously described [70].

### 4.2. Sample Preparation

Samples for metabolite profiling were collected from seedlings grown in hydroponic culture for one month. The second and third upper leaves from three individual plants per line were harvested, frozen in liquid nitrogen, and combined as one biological replicate of each sample for metabolite extraction. Three biological replicates were collected from each genotype.

Leaves for RNA extraction were collected from plants identical to those used in the metabolomic analysis. Each sample was harvested from three individual plants and frozen in liquid nitrogen.

### 4.3. Metabolite Profiling

We used a grinder (mm 400, Retsch, Haan, Germany) at 30 Hz for 1.5 min to grind samples that were freeze-dried in a vacuum. Then, 100 mg of powder was weighed, and 70% methanol aqueous solution was added at 0.1 mg/mL. The sample mixture was extracted by ultrasonication at 40 Hz for 10 min. After centrifugation and filtration (SCAA-104, 0.22 mm pore size; ANPEL, Shanghai, China, http://www.anpel.com.cn/), the metabolites in the mixture were quantified by the MRM method of LC-MS 8060 (Shimadzu, Kyoto, Japan) [48,71,72], setting the detection window to 120 s and the target scan time to 1.5 s. A total of 796 transitions were monitored, and the original data were processed by Multiquant 3.0.2. We divided the relative signal strengths of the metabolites by the strength of the internal standard (0.1 mg L^−1^ lidocaine) for normalization and then log 2 transformed the values to further improve the normalization.

### 4.4. RNA-Sequencing

Total RNA was extracted with a TRIzol reagent (Cat# DP424, TIANGEN Biotech Co. Ltd., Beijing, China) according to the protocol provided by the manufacturer. We determined the integrity of the total RNA with a 2100 Bioanalyzer (Agilent, EN, USA), which is quantified using a NanoDrop (Thermo Scientific, DE, USA). Then, we construct the sequencing library using RNA samples with high-quality (OD260/280 = 1.8 to 2.2 approximately, OD260/230 ≥ 2.0, RIN ≥ 8, > 1 μg). We purified polyA mRNA from total RNA using oligo-dT-attached magnetic beads. The purified polyA mRNA was then subjected to a fragmentation buffer. Taking these short fragments as templates, the first-strand cDNA was synthesized using reverse transcriptase and random primers, followed by second-strand cDNA synthesis. Then, the synthesized cDNA was subjected to end repair, phosphorylation, and “A” base addition according to the library construction protocol. Then, sequencing adapters were added to both sides of the cDNA fragments. After PCR amplification of the cDNA fragments, the 150 to 250 bp target fragments were cleaned up. Then, we performed paired-end sequencing on an Illumina HiSeq X Ten platform (Illumina Inc., San Diego, CA, USA).

### 4.5. RNA-Sequencing Data Analyses

To remove low-quality bases and sequencing adapters, the raw data were first processed by FASTP v0.19.4 with default settings. Subsequently, the clean data were mapped to the rice reference genome (http://rice.plantbiology.msu.edu/pub/data/Eukaryotic_Projects/o_sativa/annotation_dbs/pseudomolecules/version_7.0/all.dir/all.con) using Hisat2 v2.1.0. The conversion of the mapping output files from SAM to BAM format and the sorting by positions were performed using SAMTOOLS v1.9. StringTie v1.3.4 was used to determine the FPKM values and read counts by using the script preDE.py. DEG analysis was performed with count tables in R v3.6.2 using DEseq2, and genes with a *p*-value < 0.05 and FC > 2 were classified as DEGs.

The KEGG analysis was conducted with DEGs by using the David website (https://david.ncifcrf.gov/). For further pathway analysis of DEGs, MapMan v3.6.0 (http://mapman.gabipd.org/web/guest) was used to visualize stress-related and overview changes. We summarized an overview pathway based on MapMan results and then mapped the different expression genes to the overview pathways with MapMan software.

### 4.6. Validation of RNA-Seq Data

The relative expression of the DEGs was validated by qRT-PCR. The primers of the nominated genes were designed using Oligo7 software [73] or previously published [74,75]. The primers used in this study were given in Additional File 2: Table S7. The reactions were performed with an ABI QuantStudio 7 Flex Real-Time PCR system (Applied Biosystem, Foster City, CA, USA) using SYBR^®^ Premix Ex Taq™ II (Takara, Tokyo, Japan). Ubiquitin (LOC_Os09g39500) was used as an internal control in qRT-PCR. The relative expression of the DEGs was calculated with the 2^−ΔΔCT^ method. The qRT-PCR assay was carried out using two biological replicates with two technical replicates.

### 4.7. Phylogenetic Tree Construction

ClustalW in MEGA7 (https://www.megasoftware.net/) was used for alignment with protein sequences, and the maximum likelihood method was used to construct phylogenetic trees. After obtaining the newick file, the file was imported into the EvolView online tool (https://www.evolgenius.info//evolview/) for processing and beautification.

### 4.8. Data Availability

RNA sequence data that support the findings of this study have been deposited under SRA BioProject accession number PRJNA622884.

## Supplementary Materials

The following are available online at https://www.mdpi.com/2218-1989/10/11/425/s1, Figure S1: Correlation analysis of transcriptome data from each biological replicate of mutants and WT. Figure S2: The expression of DEGs in *d10*, *d14*, and WT. (A) Average gene expression levels of *D10* (LOC_Os01g54270), *PIN1a* (LOC_Os06g12610), and *PIN1b* (LOC_Os02g50960) in *d10*, *d14*, and WT. The error bar represents the mean value ± SD. The *p*-values were calculated using DESeq2 in R (compared with WT, * *p* < 0.05, ** *p* < 0.01). (B) qRT-PCR based expression levels of *D10*, *D14* in *d10*, *d14*, and WT. The data are represented as mean ± SD of two biological replicates. The Student’s *t*-test analysis indicates a significant difference (compared with WT, * *p* < 0.05, ** *p* < 0.01). Figure S3: Expression level of genes involved in gibberellin (GA) pathway. The error bar represents the mean value ± SD. The *p*-values were calculated using DESeq2 in R (compared with WT, * *p* < 0.05, ** *p* < 0.01). Figure S4: Expression level of genes with potential roles in lipid metabolism. The error bar represents mean value ± SD. The *p*-values were calculated using DESeq2 in R (compared with WT, * *p* < 0.05, ** *p* < 0.01). Table S1: List of the metabolites detected in this study. Table S2: The content of DAMs in each genotype. Table S3: RNA-sequencing data statistics. Table S4: FPKM of differentially expressed genes in each genotype. Table S5: Fold changes of *CPSs* and *KSs* in each mutant. Table S6: The list of putative gene clusters with altered gene expression in the mutants. Table S7: A list of DEGs primers used for RNA-sequencing data validation.

## Abbreviations

SLs: strigolactones; CCD7/8: carotenoid cleavage dioxygenase 7/8; *IPA1: Ideal Plant Architecture 1*; DAMs: differentially accumulated metabolites; DEGs: differentially expressed genes; KEGG: Kyoto Encyclopedia of Genes and Genomes; CHS: chalcone synthases; CHI: chalcone isomerases; DiTPSs: diterpene synthases; CPS: copalyl diphosphate synthase; KS: kaurene synthase; QTL: quantitative trait locus; PCs: phosphatidylcholines; lysoPCs: lysophosphatidylcholines; PCA: principal component analysis; PLD: phospholipase D; PAs: phosphatidic acids; PEAMTs: phosphoethanolamine *N*-methyltransferases.
