# Supplementary material for: Integrative Metabolomic and Transcriptomic Analyses Reveal Metabolic Changes and Its Molecular Basis in Rice Mutants of the Strigolactone Pathway
Source: Metabolites. 2020 Oct 26;10(11):425. doi: 10.3390/metabo10110425 (PMC7693813; doi:10.3390/metabo10110425)
Supplement: Supplementary file 1 [file metabolites-10-00425-s001.zip › metabolites-944774-supplementary/metabolites-944774-supplementary-proof done/Additional File 1_metabolites 20201021.pdf]

ADDITIONAL FILE 1

Integrative metabolomic and transcriptomic analyses revealed metabolic changes and its molecular basis in rice mutants of strigolactone pathway

Xiujuan Zhou[1], Ling Liu[1], Yufei Li[2], Kang Li[2], Xiaoli Liu[1], Junjie Zhou[1], Chenkun Yang[2], Xianqing Liu[1], Chuanying Fang[1,*], and Jie Luo[1,2*]

[1]College of Tropical Crops, Hainan University, Haikou, Hainan 570288, China

[2] National Key Laboratory of Crop Genetic Improvement and National Center of Plant Gene Research (Wuhan), Huazhong Agricultural University, Wuhan 430070, China

[*]Correspondence: cyfang@hainanu.edu.cn (C. Fang), jie.luo@hainanu.edu.cn (J. Luo)

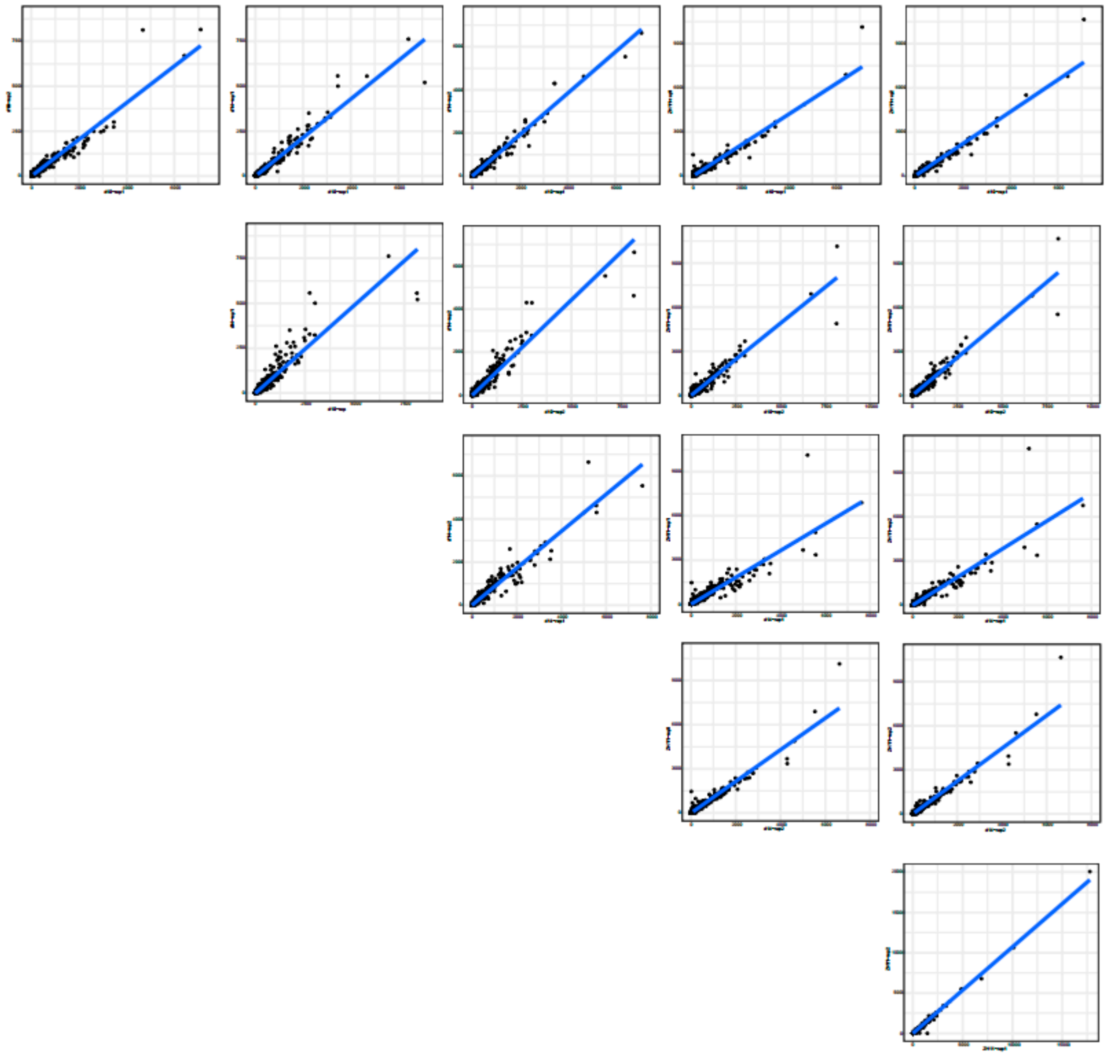

Figure S1. Correlation analysis of transcriptome data from each biological replicate of mutants and WT. The data used to plot is the FPKM value.

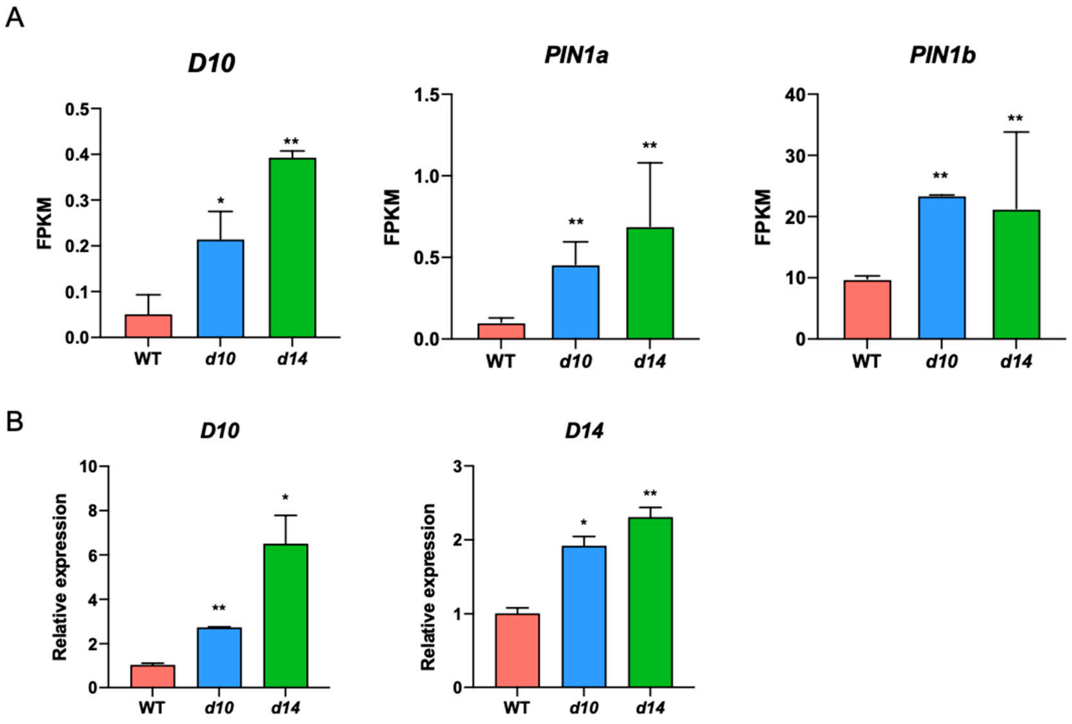

Figure S2. The expression of DEGs in *d10*, *d14* and WT. (A) Average gene expression levels of *D10* (LOC_Os01g54270), *PIN1a* (LOC_Os06g12610) and *PIN1b* (LOC_Os02g50960) in *d10*, *d14* and WT. The error bar represents mean value ± SD. The p-values were calculated using DESeq2 in R (compared with WT *$p < 0.05$, **$p < 0.01$). (B) qRT-PCR based expression levels of *D10*, *D14* in *d10, d14* and WT. The data are represented as mean ± SD of two biological replicates. The Student's t-test analysis indicates a significant difference (compared with WT, *$p < 0.05$, **$p < 0.01$).

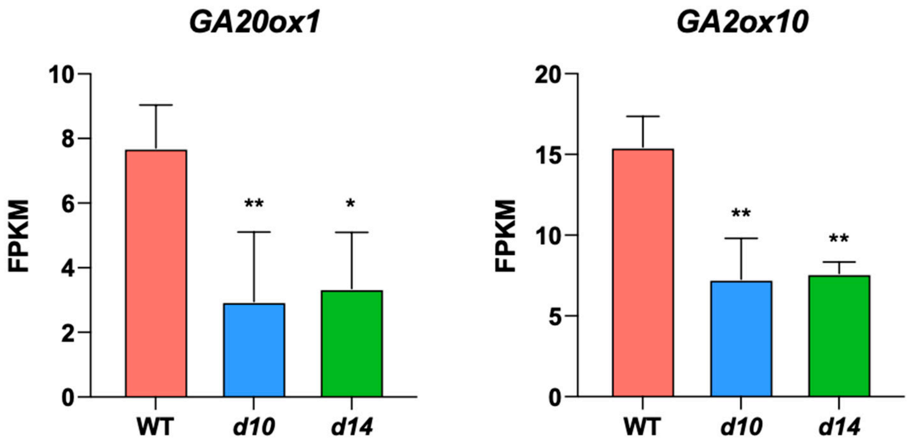

Figure S3. Expression level of genes involved in gibberellin (GA) pathway. The error bar represents mean value ± SD. The *p*-values were calculated using DESeq2 in R (compared with WT, *$p < 0.05$, **$p < 0.01$).

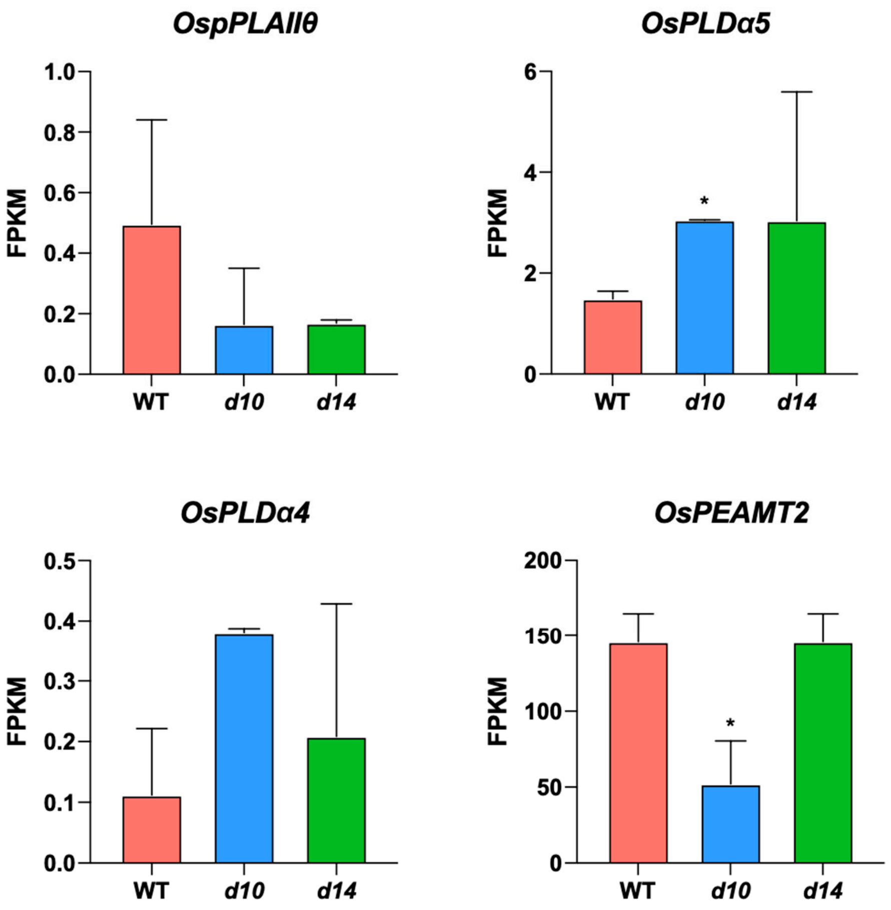

Figure S4. Expression level of genes with potential roles in lipid metabolism. The error bar represents mean value ± SD. The *p*-values were calculated using DESeq2 in R (compared with WT, *$p < 0.05$, **$p < 0.01$).
